# Longitudinal dynamics of gut microbiota and mycobiota in pneumonia-derived sepsis: evidence of taxonomic stability and trans-kingdom network reorganization

**DOI:** 10.1186/s13099-026-00838-0

**Published:** 2026-05-30

**Authors:** Fangyi Li, Xinyi Xu, Minggen Zhou, Shangming Du, Zhijie He, Zhongsheng Xia, Qikui Chen, Jieyao Li, Song Tang, Wa Zhong, Jihao Xu

**Affiliations:** 1 Department of Critical Care Medicine, Sun Yat-Sen Memorial Hospital, Sun Yat-Sen University, Guangzhou, 510120 Guangdong China; 2 Department of Gastroenterology, Beijing Friendship Hospital, State Key Laboratory of Digestive Health, Capital Medical University, Beijing, 100050 China; 3 Department of Gastroenterology, Sun Yat-Sen Memorial Hospital, Sun Yat-Sen University, Guangzhou, 510120 Guangdong China; 4 Guangdong Provincial Key Laboratory of Malignant Tumor Epigenetics and Gene Regulation, Sun Yat-Sen Memorial Hospital, Sun Yat-Sen University, Guangzhou, 510120 Guangdong China; 5 Ludwig Maximilian University of Munich, Munich, Germany; 6 Department of Gastrointestinal Surgery, The First Affiliated Hospital of Guangdong Pharmaceutical University, Guangzhou, 510080 Guangdong China

**Keywords:** pneumonia, sepsis, microbiota, mycobiota, prognosis

## Abstract

**Background:**

Gut microbial dysbiosis has been implicated in sepsis-related organ dysfunction. However, the longitudinal dynamics of the gut microbiota and mycobiota—and particularly their cross-kingdom ecological organization—in pneumonia-derived sepsis remain incompletely understood.

**Methods:**

Patients with pneumonia-derived sepsis were prospectively enrolled. Fecal samples and clinical data (SOFA scores and inflammatory markers) were collected on Day 1 and Day 7. Gut bacterial and fungal communities were profiled using 16 S rRNA and ITS1 sequencing. Longitudinal and outcome-stratified analyses were performed. Trans-kingdom co-occurrence networks and module-based topological analyses were constructed, and associations with clinical parameters were explored.

**Results:**

Global analyses indicated relative compositional stability in the gut microbiota and mycobiota between Day 1 and Day 7, with no significant differences in alpha or beta diversity. The dominant bacteria were Bacillota, Bacteroidota, and Pseudomonadota at the phylum level, and *Enterococcus*, *Bacteroides*, and *Escherichia-Shigella* at the genus level; *Escherichia-Shigella* showed a decreasing trend and *Bacteroides* an increasing trend, though neither reached statistical significance (Padj > 0.05). Ascomycota dominated the fungal community, with *Candida*, *Fusarium*, and *Oligophagozyma* as the core genera, with no obvious temporal shifts. However, outcome-based stratification revealed that fungal Chao1 richness increased significantly post-treatment, specifically in the bad-outcome group (*P* < 0.05). The most notable findings emerged from the trans-kingdom interactome. In the favorable-outcome group, a specific modular configuration (ModM1) was identified post-treatment, containing four microbiota hubs (*Parabacteroides*, *Mediterraneibacter*, *Serratia*, and *Enterococcus*). While the aggregate abundance of ModM1 lacked clinical correlation, its hub genus, *Mediterraneibacter*—a prevalent anaerobe—showed a negative association with PCT and TNF-α. Additionally, the fungal-integrated ModM8 showed a potential positive association with IL-8. Conversely, the bad-outcome group showed a lack of such hub-anchored coordination.

**Conclusions:**

In this small exploratory cohort, early pneumonia-derived sepsis appeared to exhibit relative taxonomic stability but subtle reorganization of cross-kingdom ecological connectivity. Microbial shifts appeared to manifest primarily as changes in network embedding rather than abundance. These observations provide exploratory insights that require further validation regarding topological integration, especially fungal involvement in inflammatory modules, for understanding host–microbiome interactions in critical illness. Larger longitudinal studies are warranted.

**Trial registration:**

ClinicalTrials.gov, NCT04525677, registered on 14 July 2020.

**Supplementary Information:**

The online version contains supplementary material available at 10.1186/s13099-026-00838-0.

## Introduction

Pneumonia is a severe lower respiratory tract infection often caused by bacteria, viruses, or fungi, leading to alveolar inflammation [1]. Severe pneumonia can progress to sepsis, a life-threatening condition resulting from infection-induced organ dysfunction, and studies show that pneumonia is the most common cause of sepsis[2, 3]. Despite advances in sepsis treatment improving survival rates, pneumonia-derived sepsis remains a major cause of morbidity, particularly in patients receiving mechanical ventilation[4, 5]. Therefore, further efforts in early risk stratification, precise endotyping, and the development of targeted therapeutic interventions are imperative to optimize the clinical outcomes of these patients.

The gut microbiome, composed of bacteria, fungi, and other microbes, works alongside the intestinal epithelial barrier to protect the host from pathogenic colonization. Disruption of either component can lead to microbial translocation, triggering inflammatory responses and immune reactions. Increasing evidence supports the significant role of gut microbiota in the onset, progression, and outcomes of sepsis[6–8]. Compared to healthy individuals, sepsis patients exhibit reduced microbial diversity and a disrupted flora structure, with an increase in *Enterococcus*[9–13]. A study of 150 sepsis patients revealed that low microbiota diversity is associated with a higher risk of mortality [14]. Shifts in the intestinal *Bacteroidetes* to *Firmicutes* (B/F) ratio have been linked to survival in critically ill patients [15, 16]. Both animal and human studies suggest that changes in gut microbiota composition and function contribute to sepsis-related organ damage. The gut–liver, gut-lung, gut-brain, and gut-kidney axes are frequently implicated in sepsis-induced organ injury [17–20]. For instance, via the immune system, the gut microbiome can affect the balance of the lung microbiome through the gut−lung axis [21].

Despite extensive research on gut bacteria, the fungal component of the gut microbiota—the mycobiota—has been shown to play important roles in a variety of diseases [22], yet has long been overlooked in sepsis studies. Recent cross-sectional investigations, such as gut mycobiota dysbiosis after sepsis and trauma, have shown that sepsis and trauma can induce marked perturbations in the gut fungal community, including reduced fungal diversity, loss of commensal taxa, and overgrowth of opportunistic pathogens such as *Candida spp*., persisting for up to two to three weeks after critical illness onset [23]. While these studies highlight the vulnerability of the gut mycobiota in critical illness, their reliance on single-timepoint sampling limits the understanding of temporal dynamics and inter-kingdom interactions during disease progression. In pneumonia-derived sepsis, the gut environment can change rapidly under the combined influence of infection, antibiotics, and host immune responses. Longitudinal study designs, such as those enabled by standardized intensive care unit data formats like CLIF (Common Longitudinal ICU data Format), allow repeated microbiome measurements within the same patient over time, thereby capturing dynamic microbial shifts, microbiota-fungal crosstalk, and their interactions, and facilitating the identification of microbial signatures relevant to clinical outcomes and precision interventions [24]. However, to date, comprehensive longitudinal analyses integrating both microbiota and fungal communities in pneumonia-derived sepsis remain lacking.

Given these research gaps, a comprehensive longitudinal analysis of the intestinal microbiota and fungal communities in patients with pneumonia-derived sepsis is highly warranted. In this study, we enrolled a cohort of patients with pneumonia-derived sepsis and performed longitudinal 16 S rRNA and ITS2 sequencing on stool samples collected at baseline and after treatment. By integrating clinical parameters with cross-kingdom microbiota and fungal microbial profiling, we aimed to explore the potential dynamic shifts in the gut microbiota and provide insights into its associations with disease progression and clinical outcomes.

## Materials and methods

### Study design

Patients with pneumonia-derived sepsis were enrolled from the Intensive Care Unit (ICU) of the Sun Yat-sen Memorial Hospital, Sun Yat-sen University, China, between July 2020 and February 2021. The inclusion criteria were: age between 18 and 85 years, provision of signed informed consent form, expected ICU hospital stay longer than 24 h, and confirmed diagnosis of pneumonia-derived sepsis (diagnosed with pneumonia associated with an increase of at least 2 points in the Sequential Organ Failure Assessment (SOFA) score from baseline) within 24 h [25–27]. The exclusion criteria were: pregnant or breastfeeding women, expected survival of less than 24 h, ICU stay longer than 7 days before sepsis diagnosis, sepsis originating from other or unknown sources, and when early EN within 48 h is not feasible. All enrolled patients received standardized treatment based on sepsis guidelines, including the administration of broad-spectrum antibiotics, fluid resuscitation, and organ support therapy. None of the patients received immune-related treatments, such as chemotherapy, radiotherapy, or immunomodulatory therapy. This trial was approved by the Ethics Committee of Sun Yat-sen Memorial Hospital of Sun Yat-sen University (SYSEC-KY-KS-2020-107) and registered at ClinicalTrials.gov (NCT04525677).

Patients were grouped in two ways. First, they were divided into baseline and post-treatment groups: AC (baseline) and AT (post-treatment after seven days). Second, patients were grouped by treatment outcomes based on changes in SOFA scores. Relative changes in SOFA scores were calculated as: Δ-SOFA = day1 SOFA score - day7 SOFA score. Patients were then classified according to whether Δ-SOFA represented a ≥ 25% reduction. The groups were defined as follows: BC (bad-outcome at baseline), BT (bad-outcome after seven days of treatment), GC (good-outcome at baseline), and GT (good-outcome after seven days of treatment) [28].

### Patient clinical data collection

Patients were followed up until 28 days after study enrollment. Age, sex, and diagnosis of cancer and diabetes were recorded on day 1 of inclusion. SOFA scores, Acute Physiology and Chronic Health Evaluation (APACHE II) scores, and laboratory markers such as CRP (C-Reactive Protein), procalcitonin (PCT), and plasma inflammatory cytokines (IL-6, IL-8, TNF-α, IL-1β, IL-10, and ferritin) were collected on the same days as stool samples. Other data included 28-day mortality, duration of invasive mechanical ventilation, ICU length of stay, and isolated pathogenic bacteria and antibiotics use.

### Stool collection

Stool samples were collected on day 1 and 7 after inclusion. A sample was taken from a deep part of the fresh stool using a sampling spoon, quickly placed in a standard stool collection tube and transferred in liquid nitrogen. Prior to genomic DNA extraction, stool samples were stored at -80℃.

### Stool DNA extraction and sequencing

Total genome DNA from samples was extracted using the CTAB method[29]. DNA concentration and purity were monitored using 1% agarose gels. DNA was diluted to 1 ng/µL using sterile water. For the DNA sample preparations, 1 µg DNA per sample was used. DNA fragments were end-polished, A-tailed, and ligated with the full-length adaptor for Illumina sequencing, followed by PCR amplification. PCR products were purified (AMPure XP system) and libraries were analyzed for size distribution by Agilent 2100 Bioanalyzer and quantified via real-time PCR. Cluster generation was performed using the cBot Cluster Generation System and sequencing was conducted on the Illumina NovaSeq 6000 platform to generate paired-end reads.

For microbiota community profiling, amplicon sequencing of the 16S rRNA gene V3-V4 hypervariable region was performed using primers 341F (5’-CCTAYGGGRBGCASCAG-3’) and 806R (5’-GGACTACNNGGGTATCTAAT-3’). For fungal community profiling, amplicon sequencing targeted the ITS1 region, using primers ITS1-1 F-F (5’-CTTGGTCATTTAGAGGAAGTAA-3’) and ITS1-1 F-R (5’-GCTGCGTTCTTCATCGATGC-3’).

### Sequence data processing

Raw paired-end sequencing reads were processed using QIIME2 (v2025.4). Demultiplexed sequences were quality filtered, trimmed, merged, and denoised using the DADA2 plugin, which includes error modeling, dereplication, chimera removal, and inference of exact amplicon sequence variants (ASVs). This denoising strategy avoids arbitrary similarity-based clustering and provides higher taxonomic resolution, improved reproducibility, and reduced false-positive sequence variants compared with traditional OTU-based approaches. To evaluate whether the sequencing depth was sufficient to capture the majority of microbial diversity, rarefaction curves were generated for all samples. The observed plateaus in these curves indicated that the sequencing effort was adequate for subsequent diversity estimation.

Representative ASV sequences were used for downstream taxonomic and phylogenetic analyses. For microbiota community profiling, ASVs derived from the 16 S rRNA gene were taxonomically classified using a Naive Bayes classifier implemented in the QIIME2 classify-sklearn plugin and trained on the SILVA reference database (v138.2). For fungal community profiling, ITS-derived ASVs were classified against the UNITE database (v 10.0). Taxonomic abundance tables were generated at multiple hierarchical levels, including phylum, class, order, family, genus, and species, with subsequent analyses primarily focused on the phylum- and genus-level profiles.

Multiple sequence alignment of ASV representative sequences was performed, and a phylogenetic tree was constructed to support phylogeny-based community analyses. Alpha diversity metrics were calculated to characterize within-sample microbial richness and evenness, while beta diversity analyses were conducted to assess between-sample community dissimilarity. Hierarchical clustering of samples based on beta diversity distances was additionally performed to illustrate patterns of microbial similarity among samples.

To reduce data sparsity and improve statistical robustness, ASVs were filtered prior to downstream analyses based on the following criteria: ASVs were retained only if they had a relative abundance > 0.01% and were detected in at least three samples with a total read count of no less than 20. In addition, ASVs assigned to mitochondria, chloroplasts, or those unclassified at the phylum level were excluded. Relative abundance tables were generated using total sum scaling (TSS) normalization, and log-transformation was applied where appropriate for statistical modeling.

### Statistical analysis

Clinical data were analyzed using R (v4.5.2), with continuous variables compared via Student’s t-tests and categorical data via the χ^2^ test.

Microbial diversity was assessed using the “vegan” package. To account for uneven sequencing depth, ASV tables were rarefied to the minimum library size across all samples. These rarefied data were used exclusively for alpha diversity, beta diversity, and co-occurrence network analyses. Alpha diversity metrics were compared using Wilcoxon signed-rank tests to account for the paired longitudinal design. Beta diversity was evaluated based on Bray-Curtis distances and visualized via Principal Coordinate Analysis (PCoA). Structural differences were tested by Permutational Multivariate Analysis of Variance (PERMANOVA) with 999 permutations, constrained within individual participants.

Longitudinal microbial shifts were identified using ALDEx2 with centered log-ratio (CLR) transformation. Repeated-measures correlations (rmcorr) were assessed via the “rmcorr” package. Co-occurrence networks were constructed based on Spearman’s rank correlation (|r| > 0.8) and partitioned into modules using the Louvain algorithm. The topological role of each node was determined by its within-module connectivity (Zi) and among-module connectivity (Pi). Specifically, nodes were categorized into: peripherals (Zi ≤ 2.5, Pi ≤ 0.62), connectors (Zi ≤ 2.5, Pi > 0.62), module hubs (Zi > 2.5, Pi ≤ 0.62), and network hubs (Zi > 2.5, Pi > 0.62). Network visualization was performed using Gephi (v0.10.1).

Keystone taxa were identified in MATLAB (vR2024b) using a top-down framework based on Expected Positive Influence (EPI) metrics. We evaluated three dimensions of network influence: D1, representing direct impact on immediate neighbors; D2, capturing indirect ‘ripple effects’ through secondary connections; and Q, reflecting overall topological importance. Taxa were defined as keystone candidates only if all three EPI metrics exceeded the mean by two standard deviations (mean + 2SD), which evaluate a taxon’s contribution to community stability through its positive associations [30].

Notably, Benjamini-Hochberg (BH) correction was strictly applied to ALDEx2 and network analyses, with an adjusted *P* (*Padj*) < 0.05 considered statistically significant. For other tests, a raw *P* < 0.05 was used as the significance threshold.

## Results

### Characteristics of patients

A total of 28 fecal samples were obtained from 14 patients with pneumonia-derived sepsis (mean age, 65 years; 57% male) on Day 1 and Day 7 after enrollment. Detailed patient characteristics are presented in Table 1, and baseline enrollment information is provided in Supplementary Fig. 1. ICU length of stay was significantly longer in patients with poor outcomes (*P* < 0.05). Among the 14 patients with confirmed pneumonia, 6 pathogens were detected via bronchoalveolar lavage fluid (BALF) culture, with 2 confirmed by BALF metagenomic next-generation sequencing (mNGS). Only 1 pathogen was identified through blood mNGS and no multidrug-resistant organisms (MDROs) were found. Most patients received carbapenems combined with glycopeptide antibiotics, with no antifungal drug use during the 7-day study period (Supplementary Fig. 2).

**Table 1:** Clinical factors of sepsis hospitalizations

Characteristics	Bad-outcome group (*n* = 7)	Good-outcome group (*n* = 7)	*P*-value
Age (years), mean (SD)	66.29 (13.34)	63.71 (9.71)	0.565
Male, n (%)	4 (57.14)	4 (57.14)	1
Weight^a^, mean (SD)	60.29 (20.99)	61.89 (13.96)	0.814
Cancer, n (%)	3 (42.86)	2 (28.57)	0.693
Diabetes, n (%)	2 (28.57)	4 (57.14)	0.252
APACHE II^b^ day1, mean (SD)	27.86 (4.56)	28.71 (3.55)	0.702
APACHE II day7, mean (SD)	24.00 (6.48)	23.86 (4.14)	0.962
SOFA^c^ day1, mean (SD)	8.00 (2.77)	9.43 (3.41)	0.406
SOFA day7, mean (SD)	7.29 (2.50)	5.86 (2.04)	0.264
ICU length of stay(days), mean (SD)	21.86 (8.93)	12.00 (6.08)	0.002
Duration of mechanical ventilation, mean (SD)	18.86 (9.89)	9.29 (6.52)	0.054
28-day mortality^d^, n (%)	1 (14.29)	1 (14.29)	1

This table describes the baseline characteristics of septic patients. Significant group differences were determined by using Student t-tests for continuous variables and χ^2^ for categorical variables

^a^ patient’s weight on the inclusion day

^b^Acute Physiology and Chronic Health Evaluation II score

^c^Sequential Organ Failure Assessment score

^d^death occurs within 28 days of inclusion

### Comparable gut microbiota diversity and composition between two time points in pneumonia-derived sepsis

**Fig. 1:**
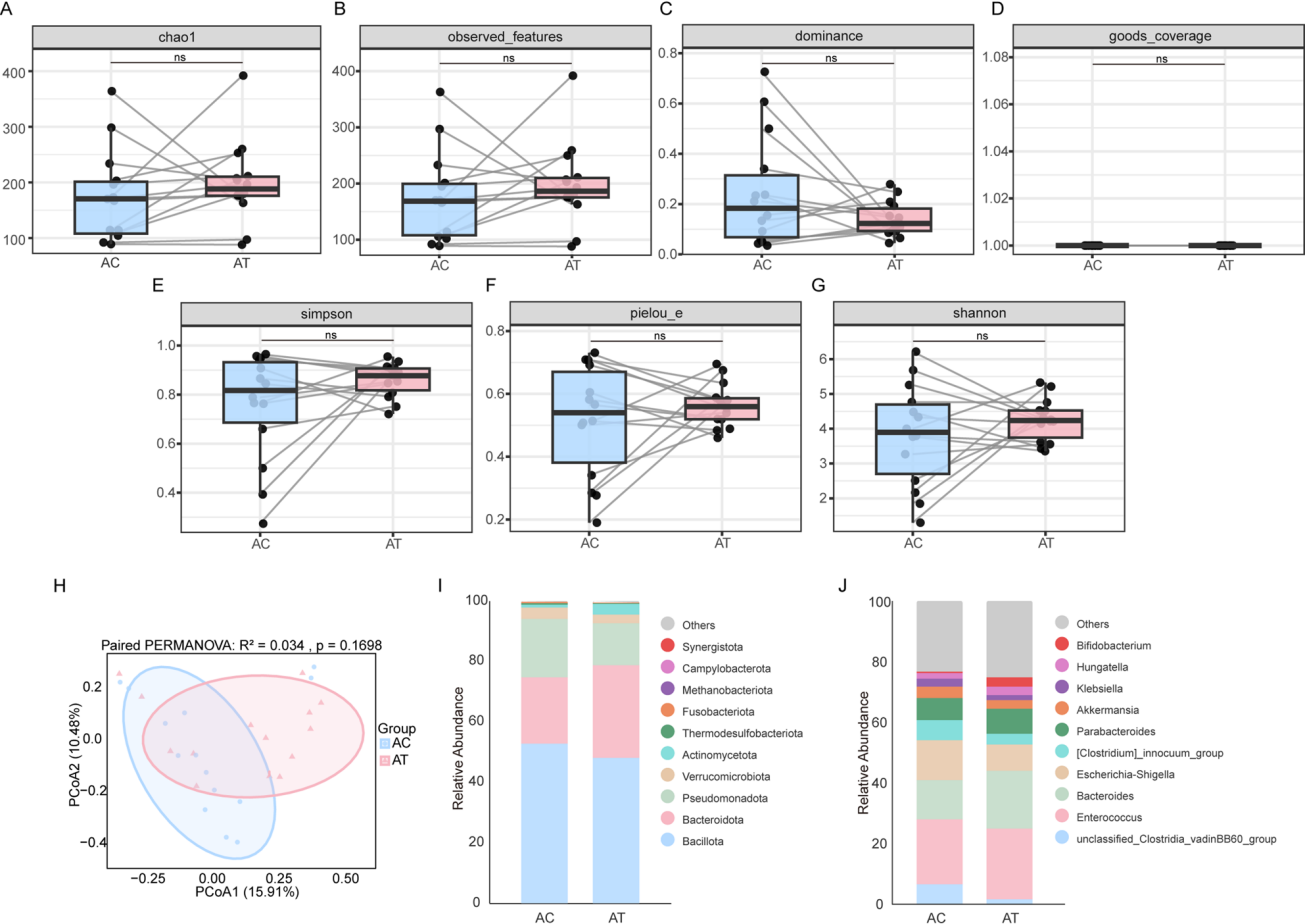
Dynamics and stability of the gut microbiota throughout the treatment period. **(A–G)** Assessment of gut microbiota alpha-diversity at Day 1 (AC) and Day 7 (AT). Indices include richness (Chao1, Observed features), evenness (Pielou’s), and overall diversity/dominance (Simpson, Shannon, Dominance, and Good’s coverage). **(H)** Beta-diversity analysis visualized via Principal Coordinates Analysis (PCoA) based on Bray-Curtis distance, comparing the microbiota community structure between AC and AT. **(I–J)** Taxonomic composition of the gut microbiota. Comparison of the relative abundance of dominant microbiota phyla (I) and the most abundant genera (J) between Day 1 (AC) and Day 7 (AT). AC, baseline (Day 1); AT, post-treatment after seven days (Day7)

We analyzed the composition of fecal bacteria using 16 S sequencing. Alpha diversity (Fig. 1A and G) and beta diversity (Fig. 1H) did not significantly differ between Day 1 (AC) and Day 7 (AT). *Bacillota*, *Bacteroidota*, and *Pseudomonadota* were the predominant microbiota phyla in both groups (Fig. 1I). *Enterococcus*, *Bacteroides*, and *Escherichia-Shigella* were the dominant genera (Fig. 1J), and the relative abundance of *Escherichia-Shigella* tended to decrease while that of *Bacteroides* tended to increase after treatment. To evaluate the temporal dynamics of the gut microbiota structure, we performed differential abundance analysis at both the ASV and genus levels using ALDEx2. Despite the longitudinal tracking of the cohort, the gut microbiota exhibited high compositional stability. No microbiota taxa were identified as significantly differentially abundant (either |effect size| < 1 or *Padj* > 0.05) between the two timepoints.

### Comparable gut mycobiota diversity and composition between two time points in pneumonia-derived sepsis

**Fig. 2:**
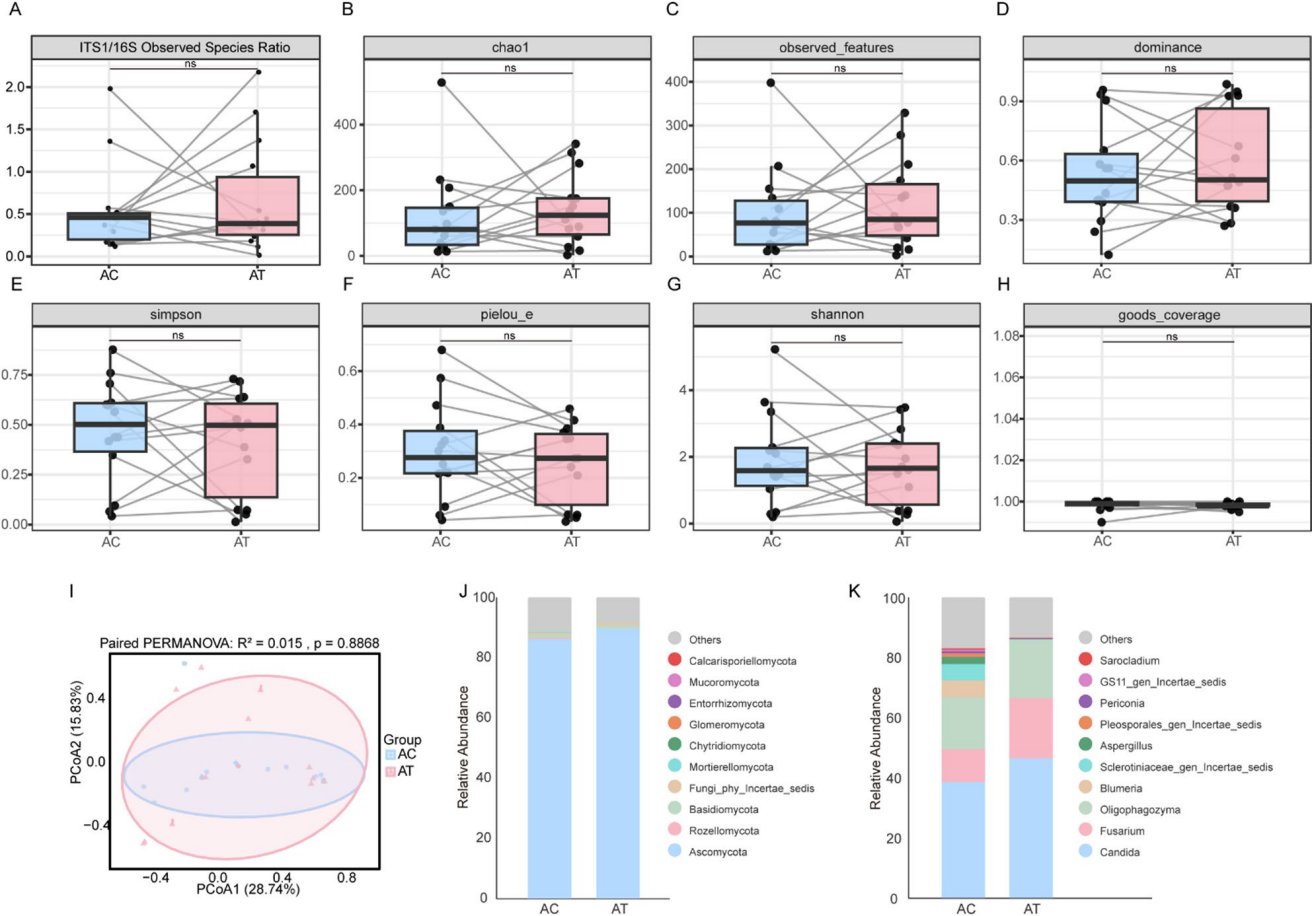
Dynamics and stability of the gut mycobiota throughout the treatment period. **(A)** Fungal-to-microbiota richness ratio. Comparison of the ratio of fungal to microbiota observed features (ITS1/16S ratio) between Day 1 (AC) and Day 7 (AT). **(B–H)** Assessment of gut fungal alpha-diversity at Day 1 (AC) and Day 7 (AT). Indices include richness (Chao1, Observed features), evenness (Pielou’s), and overall diversity/dominance (Simpson, Shannon, Dominance, and Good’s coverage). **(I)** Beta-diversity analysis visualized via Principal Coordinates Analysis (PCoA) based on Bray-Curtis distance, comparing the fungal community structure between AC and AT groups. **(J–K)** Taxonomic composition of the gut mycobiota. Comparison of the relative abundance of dominant fungal phyla (J) and the most abundant genera (K) between Day 1 (AC) and Day 7 (AT). AC, baseline (Day 1); AT, post-treatment after seven days (Day7)

We then assessed the gut fungal composition using ITS1 sequencing. The ratio of ITS to 16 S observed features showed no significant change before and after treatment (Fig. 2A). The alpha diversity (Fig. 2B and H) and beta diversity (Fig. 2I) analyses also showed no significant differences between the AC and AT groups. *Ascomycota* was the dominant fungal phylum (Fig. 2J). *Candida*, *Fusarium*, and *Oligophagozyma* were the dominant genera in both groups (Fig. 2K). Differential abundance analysis via ALDEx2 yielded no statistically significant shifts at either the ASV or genus levels.

### Comparative microbial co-occurrence networks across time points in pneumonia-derived sepsis

**Fig. 3:**
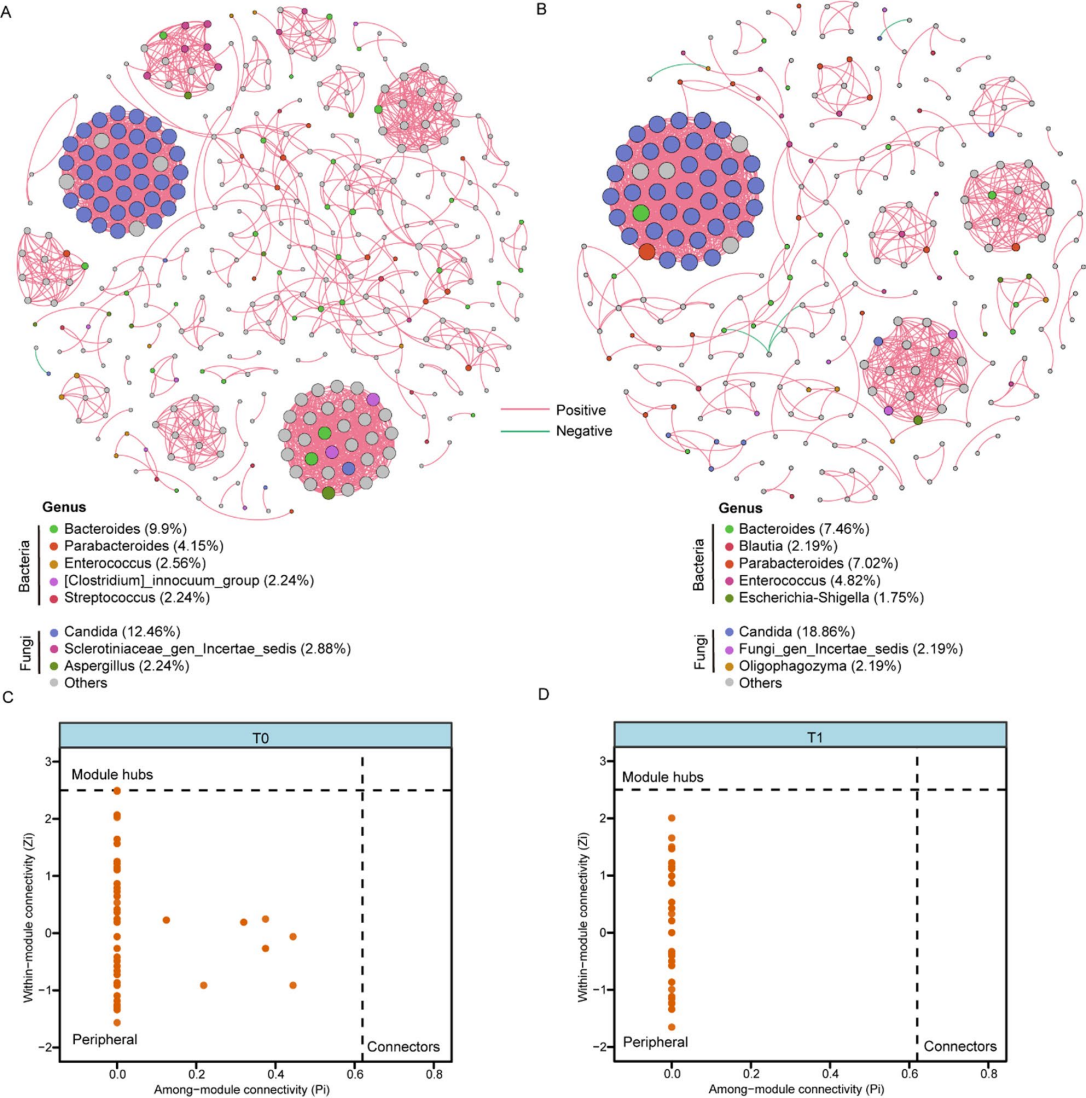
Structural and topological stability of microbial co-occurrence networks throughout the treatment period. **(A–B)** Microbial co-occurrence networks at the genus level. Networks were constructed to visualize the inter-generic interactions at Day 1 (A) and Day 7 (B). Each node represents a microbial genus, and an edge indicates a significant and robust correlation (Spearman’s |r| > 0.8, *P*adj < 0.05). Nodes are color-coded according to their taxonomic identity at the genus level. **(C–D)** Zi-Pi plots for topological role identification. Distribution of individual genera based on their within-module connectivity (Zi) and among-module connectivity (Pi) at Day 1 (C) and Day 7 (B). Horizontal and vertical dashed lines indicate the thresholds (Zi = 2.5, Pi = 0.62) used to identify potential keystone taxa, categorizing nodes into module hubs (Zi > 2.5), connectors (Pi > 0.62), and peripheral nodes

To explore potential alterations in microbial inter-relationships, we performed co-occurrence network analysis at the genus level for both the microbiota and mycobiota. Consistent with the observed taxonomic constancy, the overall topological structure of the microbial networks remained largely conserved post-treatment. No significant divergence was detected in key network metrics, including node degree distribution and clustering coefficients (Supplementary Table 3). While the global topological metrics remained stable, several qualitative transitions in the co-occurrence landscape were discernible between Day 1 and Day 7. At baseline (Day 1), the microbial network exhibited a decentralized architecture, characterized by multiple discrete clusters, each comprising a diverse assemblage of microbial genera, including both common and unassigned taxa (Fig. 3A). By Day 7, a notable contraction in microbial connectivity was observed, leading to the dissolution or weakening of these multi-taxa microbial consortia (Fig. 3B). Although no module hubs were identified in either stage, there was a clear trend toward reduced modularity (Zi) and decreased among-module connectivity (Pi) over time, suggesting a potential decline in the complexity of network interactions (Fig. 3C and D).

In contrast, the fungal component maintained a highly interconnected sub-network centered on *Candida* throughout the study period (Fig. 3A and B).

### Distinct gut microbial signature in pneumonia-derived septic patients with different outcomes

Despite the absence of global shifts, we speculated that the profound clinical heterogeneity inherent in critically ill patients—a pervasive confounding factor in critical care research—might have obscured distinct microbial signatures. Consequently, we stratified the enrolled patients into a good-outcome (G) group (baseline control, GC; after seven days of treatment, GT) and a bad-outcome (B) group (baseline control, BC; after seven days of treatment, BT) to further explore the potential associations between microbial dynamics and clinical outcomes.

**Fig. 4:**
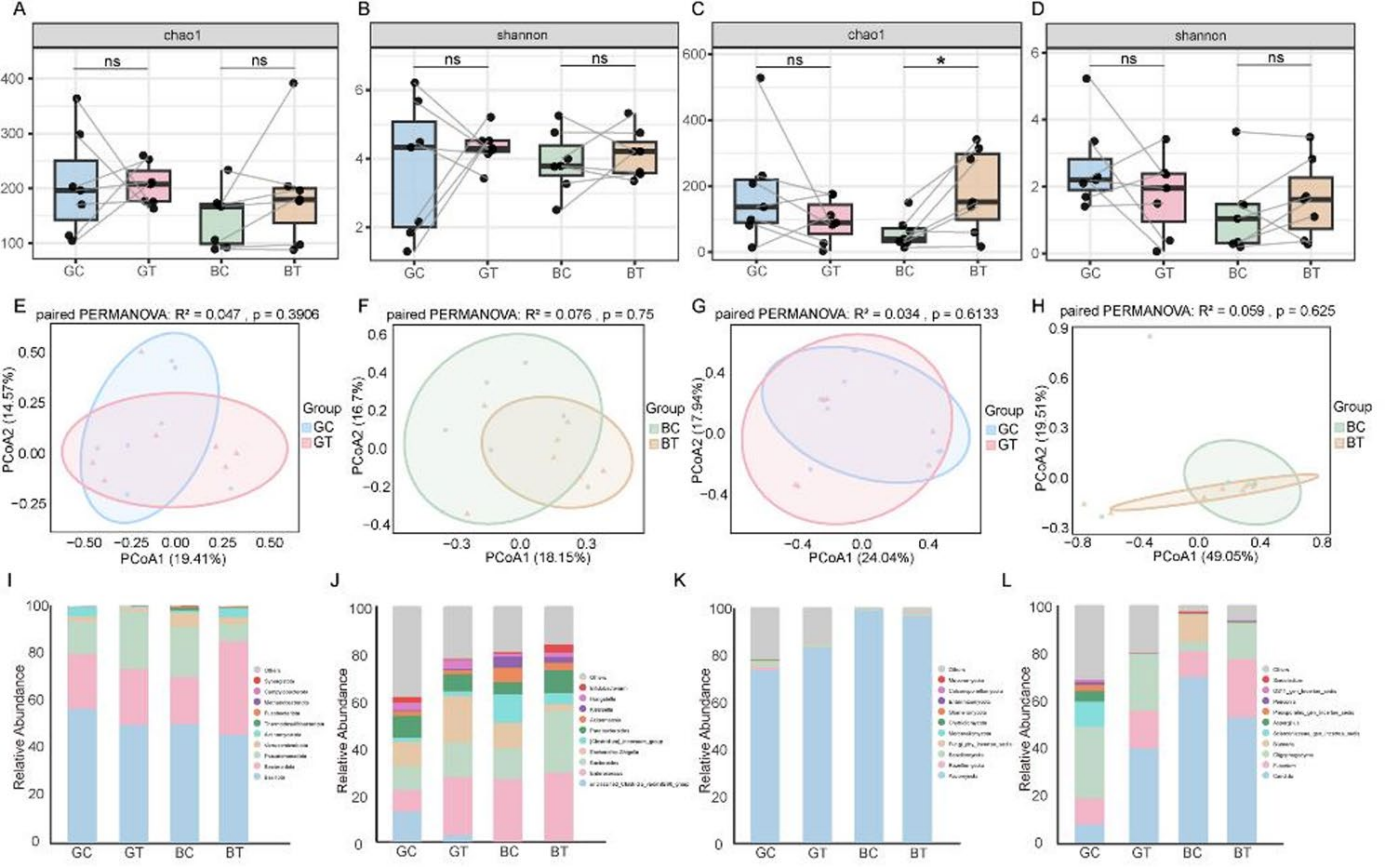
Distinct gut microbiota features stratified by clinical outcome. **(A–D)** Stratified analysis of gut microbial alpha-diversity. Comparison of Chao1 and Shannon indices for the microbiota (A, B) and mycobiota (C, D) between Day 1 and Day 7, stratified by outcome (Good-outcome: GC vs. GT; Bad-outcome: BC vs. BT). **(E–H)** Beta-diversity analysis visualized by PCoA. Principal Coordinates Analysis based on Bray-Curtis distance in good-outcome (E) and bad-outcome (F) groups for the microbiota, and good-outcome (G) and bad-outcome (H) groups for the mycobiota. **(I–L)** Taxonomic composition of the gut microbial. Comparison of the relative abundance of dominant microbiota phyla (I) and the most abundant genera (J) across outcome groups and time points. Comparison of the relative abundance of dominant fungal phyla (K) and the most abundant genera (L) across outcome groups and time points. BC, bad-outcome at baseline; BT, bad-outcome after seven days of treatment; GC, good-outcome at baseline; GT, good-outcome after seven days of treatment

Although microbiota alpha diversity showed no significant changes before and after treatment across different outcome groups (Fig. 4A and Supplementary Fig. 3A–3 F), fungal alpha diversity measured by Chao1 was significantly increased after treatment in the bad-outcome group (BC vs. BT) (Fig. 4B and Supplementary Fig. 3G–3 L), while other alpha diversity indices, such as Shannon and Simpson, remained unchanged, suggesting an increase primarily at the level of rare fungal taxa. Beta diversity of bacteria (Fig. 4C and D) and fungi (Fig. 4E and F) did not show significant differences before and after treatment in either outcome group (GC vs. GT and BC vs. BT).

At the microbiota level, the phylum *Bacteroidota* and the genus *Bacteroides* increased after treatment in the bad-outcome group (Fig. 4G and H), whereas no notable changes were observed in the good-outcome group. At the fungal level, the phylum *Ascomycota* and the genus *Candida* increased in patients with good outcomes but decreased in those with poor outcomes (GC vs. GT and BC vs. BT; Fig. 4I and J). Despite these opposite directional trends, the global pattern was mirrored by the compositional statistics, consistent with the overall community analysis. ALDEx2-based differential abundance testing detected no statistically significant changes at either the ASV or genus levels in both the good-outcome (GC vs. GT) and bad-outcome (BC vs. BT) comparisons.

Although an increased Chao1 index was observed in the poor prognosis group, further analysis revealed no significant correlations between longitudinal changes in Chao1 (from BC to BT) and clinical metrics (Supplementary Table 1). Similarly, no evident associations were found between Chao1 richness and clinical parameters, including SOFA scores and inflammatory markers within the BT group (all *P* > 0.05; Supplementary Table 2).

### Microbiota network dynamics and hub-driven modular shifts across outcome groups in the co-occurrence network

To further investigate whether interspecies relationships were contingent upon clinical outcomes, we performed stratified inter-kingdom co-occurrence network analyses at the microbiota and fungal genus level across the prognostic subgroups. Across all four groups, positive correlations dominated the co-occurrence networks.

**Fig. 5:**
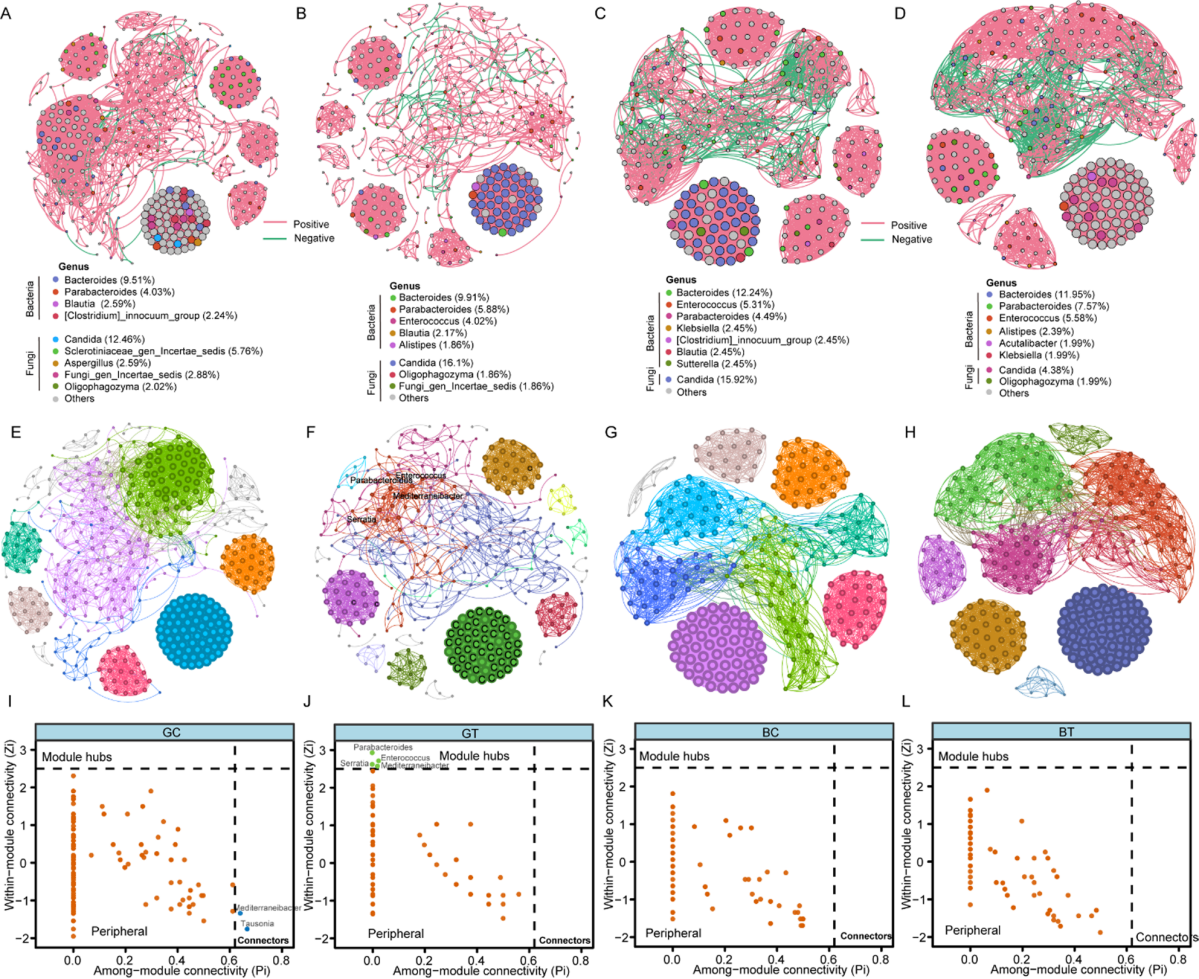
Distinct microbial co-occurrence patterns stratified by clinical outcome. **(A–D)** Outcome-stratified microbiota and fungal co-occurrence networks. Genus-level microbial networks were independently constructed for the good-outcome (A; B) and bad-outcome (C; D) groups. Nodes represent microbiota or fungal genera, and edges indicate robust and significant correlations (Spearman’s |r| > 0.8, *P*adj < 0.05). Edge colors denote positive (red) or negative (green) correlations. Nodes are color-coded according to their taxonomic identity at the genus level. **(E–H)** Modular organization of inter-kingdom networks. Nodes are partitioned into modules indicated by distinct colors, representing clusters of highly interconnected taxa. In the post-treatment good-outcome group (GT), labels highlight the four module hubs identified within the ModM1 module. **(I–L)** Classification of topological roles via Zi-Pi plots. Distribution of microbial genera based on their within-module connectivity (Zi) and among-module connectivity (Pi) for the good-outcome (I; J) and bad-outcome (K; L) groups. odes are colored by their respective modular partitions. Horizontal and vertical dashed lines indicate the thresholds (Zi = 2.5, Pi = 0.62) used to identify potential keystone taxa, categorizing nodes into module hubs (Zi > 2.5), connectors (Pi > 0.62), and peripheral nodes. BC, bad-outcome at baseline; BT, bad-outcome after seven days of treatment; GC, good-outcome at baseline; GT, good-outcome after seven days of treatment

At baseline, the good-outcome group (GC) network was characterized by several small microbiota clusters with distinct boundaries. Following treatment (GT), overall connectivity and inter-cluster links decreased (Fig. 5A and B). Conversely, in the poor-outcome groups (BC and BT), both positive and negative correlations increased, leading to higher connectivity between clusters and less distinct cluster boundaries (Fig. 5C and D). Quantitative analysis of network topological properties supported these observations. Compared with GC, the GT group showed reductions in node and edge numbers, alongside slight increases in clustering coefficient and modularity. In the BT group, total connections increased relative to BC, but network density and connectance were lower, accompanied by a longer average path length and decreased modularity (Supplementary Fig. 4A–4 N). The ratio of interdomain (bacteria-fungi) interactions was significantly elevated in the BT group compared to BC (*P* < 0.05, Supplementary Fig. 4L), suggesting a more integrated but less modular cross-kingdom community structure in patients with poor outcomes.

Given the prevalent formation of clustered structures observed in the networks described above, we next performed a module-level analysis to further investigate the organization and connectivity within the co-occurrence framework. Based on the Zi-Pi distribution, the good prognosis group showed a more complex network topology, characterized by the presence of connector genera in GC and four module hubs in GT (*Parabacteroides*, *Mediterraneibacter*, *Serratia*, and *Enterococcus*) (Fig. 5I and J). This emergence of module hubs in the GT group stands in sharp contrast to its significant reductions in global connectivity, including Mean Degree, Mean Betweenness, and Interdomain Ratio, compared to the GC group (Supplementary Fig. 4L–4 N). Conversely, such key nodes were not observed in the poor prognosis group, where all genera remained as peripherals (Fig. 5J and K).

**Fig. 6:**
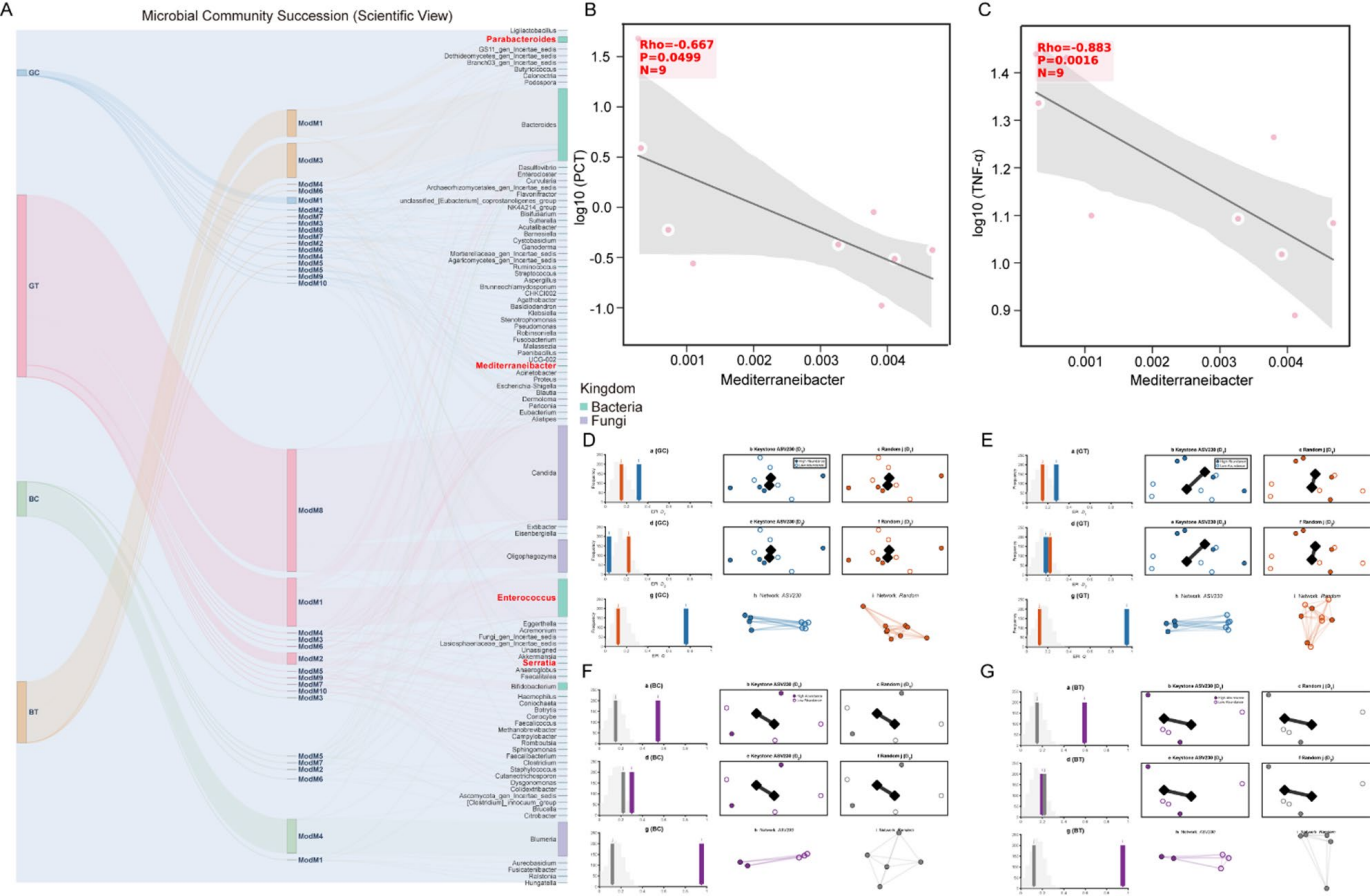
Cross-kingdom microbial interactions and their clinical relevance stratified by clinical outcome. **(A)** Sankey diagram illustrating transitions and distributions of microbial modules across outcome groups (GC, GT, BC, and BT). Modules are defined based on co-occurrence network modularity analysis. **(B-C)** Correlations between the relative abundance of *Mediterraneibacter* and clinical parameters PCT(B) and TNF-α levels(C). **(D–G)** Evaluation of the global network influence of *Mediterraneibacter* via Expected Positive Influence (EPI) analysis. Panels D–G correspond to the GC, GT, BC, and BT groups, respectively. Within each group’s assessment: (a, d,g) EPI Metric Distributions: Sub-panels display the score distributions of D1(a), D2(d), and Q (g) across all ASVs. Arrows with superscript j highlight the topological position of *Mediterraneibacter*, while arrows with superscript i indicate a random reference species. (b–f) Community Impact Ordination: Community Impact Ordination: PCoA plots based on Bray-Curtis dissimilarity illustrate the sample clustering (presence-impact effect) associated with *Mediterraneibacter*(b, e) compared to a random taxon (c, f). Black crosses denote group centroids. (h, i) Sample-level Association Networks: Sample-to-sample correlation networks centered on the candidate keystone taxon (h) versus a reference taxon (i). Filled and open nodes represent high and low abundance of the respective taxon. BC, bad-outcome at baseline; BT, bad-outcome after seven days of treatment; GC, good-outcome at baseline; GT, good-outcome after seven days of treatment

The modularity analysis of the cross-kingdom co-occurrence network, as visualized through the Sankey trajectory, identified a highly structured and specialized community configuration unique to the GT group. This group was characterized by the consolidation of the ModM1 module, where all four aforementioned hub taxa (*Parabacteroides*, *Mediterraneibacter*, *Serratia*, and *Enterococcus*) were centrally clustered (Figs. 5F and 6A). While other experimental groups (BC, BT, and GC) exhibited distinct modular distributions—such as the transition toward ModM4 or dispersed allocations across ModM2 and ModM6—they lacked the specific hub-driven coordination centered around these four key genera observed in the GT cohort (Figs. 5E and H and 6A).

To explore the potential clinical relevance of the identified hub taxa from a broader perspective, we examined the correlations between their total relative abundances—calculated across the entire cohort (including the BC, BT, GC, and GT groups) without regard to modular roles—and clinical parameters, such as SOFA scores and inflammatory markers. This pan-cohort, bulk-level assessment was designed to determine whether these specific genera possess intrinsic associations with clinical status that transcend their group-specific topological positions or localized network roles. No significant associations were found, suggesting these taxa may only influence the host when integrated within a specific modular structure (Supplementary Fig. 5).

Building upon these observations, we first assessed the correlation between the overall relative abundance of the ModM1 module and clinical indices in the GT group, which yielded no significant associations. To further resolve potential taxon-specific contributions within this structure, we performed a secondary assessment focusing exclusively on the relative abundance of the four hub genera in their specific capacities as ‘module hubs’ within ModM1. Notably, this analysis excluded any abundance contributions from these genera when acting in other topological roles or within different modules. Intriguingly, while the module as a whole and other hub members remained clinically neutral, the relative abundance of *Mediterraneibacter*—specifically when functioning as a module hub—demonstrated a significant and strong negative correlation with inflammatory markers, specifically PCT and TNF-a (Fig. 6B and C and Supplementary Fig. 6).

To further clarify whether *Mediterraneibacter* exerts its influence at a global network level or within a localized modular scale, we performed keystone taxa identification based on Expected Positive Influence (EPI). The results demonstrated that the EPI metrics (D1, D2, and Q) for *Mediterraneibacter* remained relatively stable across all four groups (GC, GT, BC, and BT), showing no significant shifts in global influence and failing to reach the candidate keystone threshold (mean + 2SD) (Fig. 6D and G). This indicates that the role transition of *Mediterraneibacter* as a hub genus is likely independent of global network fluctuations, with its impact being primarily confined to specific modular structures. These findings are consistent with the aforementioned result the total relative abundance of *Mediterraneibacter* at the global, pan-cohort level shows no significant correlation with clinical parameters (Supplemental Fig. 5).

### Fungal network dynamics and peripheral roles across outcome groups in the co-occurrence network

**Fig. 7:**
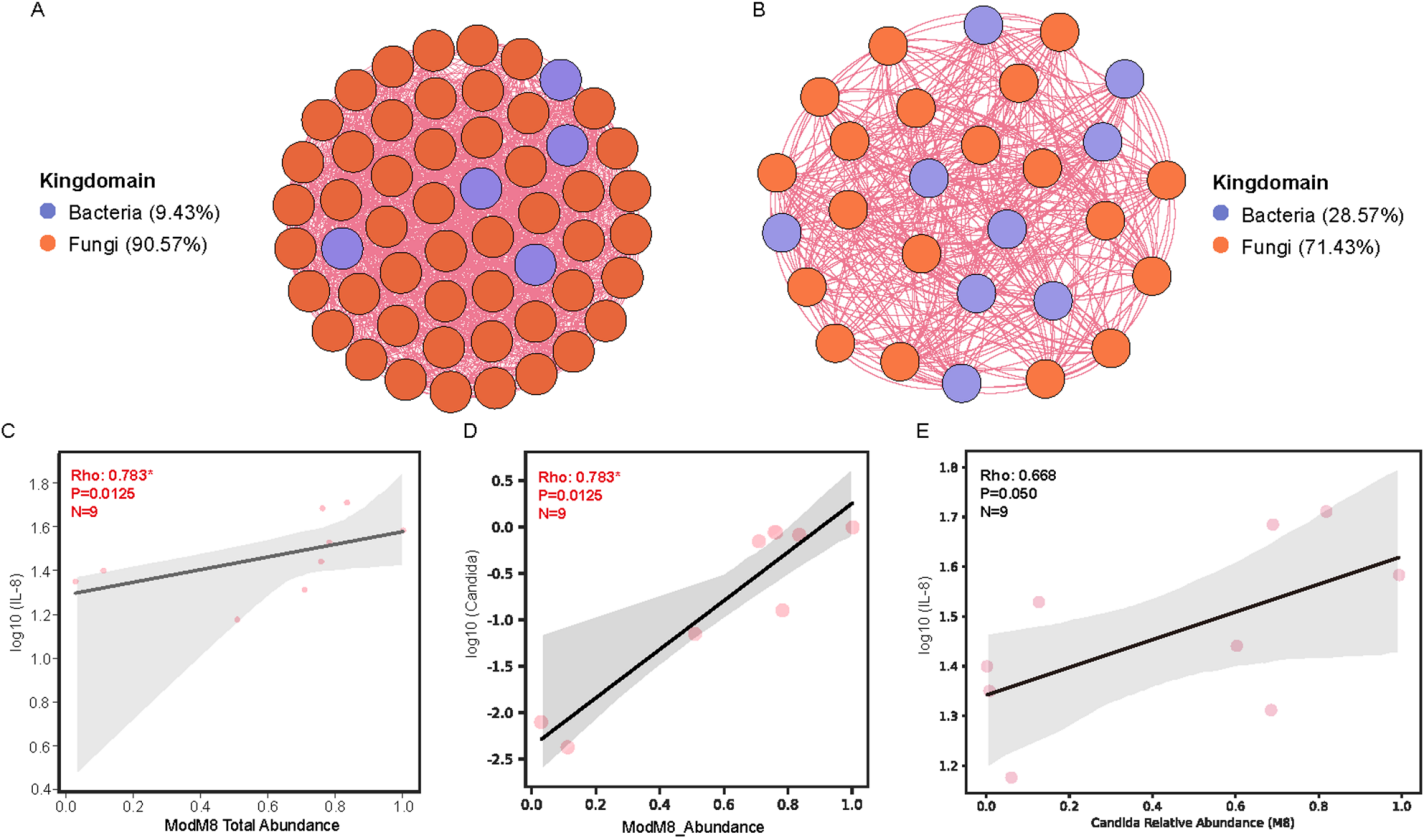
Cross-kingdom interaction patterns and clinical associations of fungal-dominated modules. **(A–B)** Composition of fungal-integrated cross-kingdom modules. ModM8 module in the GT group: A cross-kingdom assembly featuring a co-occurrence core between *Candida* and specific microbiota genera(A). ModM4 module in the BC group: An inter-kingdom structure with *Blumeria* as a central component(B). Nodes represent microbiota or fungal genera and are color-coded by kingdom to highlight trans-kingdom connectivity. **(C)** Clinical correlation of ModM8. Spearman correlation between the aggregate relative abundance of the ModM8 module and plasma IL-8 levels in the GT group. **(D)** Modular integration of *Candida*. Correlation between the total abundance of the ModM8 module and the relative abundance of the intra-modular *Candida* in the GT group, reflecting its integration within the modular structure. **(E)** Intra-modular genus-level correlation. Correlation between the relative abundance of intra-modular *Candida* (within ModM8) and plasma IL-8 levels in the GT group. BC, bad-outcome at baseline; GT, good-outcome after seven days of treatment

In the integrated microbiota–fungal co-occurrence network, fungal taxa formed a relatively independent subnetwork. Although no fungal taxa were identified as module hubs (Fig. 5E and H), *Candida* consistently exhibited a tendency to serve as a central node across groups (Fig. 5A and D). Consistent with the previously observed increase in Chao1 richness in the BT group relative to BC, the *Candida*-centered cluster in BT was markedly weakened and accompanied by the incorporation of several low-abundance fungal taxa into the network. Modular analysis further revealed two fungi-centric cross-kingdom modules. In the GT group, module ModM8 consisted of a cross-kingdom core with *Candida* and microbiota genera in co-occurrence. Similarly, in the BC group, module ModM4 was an inter-kingdom assembly featuring *Blumeria* as a central component (Figs. 6A and 7A and B).

We further analyzed the relationship between the abundance of these modules/taxa and clinical parameters, including SOFA scores and inflammatory markers. In the GT group, the total abundance of the ModM8 module demonstrated a strong positive correlation with IL-8 level (Fig. 7C and Supplementary Fig. 7). Despite a strong positive correlation between the intra-modular relative abundance of *Candida* (a core member of ModM8) and the overall ModM8 modular abundance (Fig. 7D), no significant associations were observed between this intra-modular *Candida* signal and SOFA scores or inflammatory markers within the GT group (Fig. 7E and Supplementary Fig. 8). In the BC group, neither the total abundance of the ModM4 module nor the relative abundance of *Blumeria* within ModM4 showed any statistically significant correlation with SOFA scores or inflammatory markers (Supplementary Tables 4 and Supplementary Table 5).

## Discussion

In this longitudinal prospective exploratory study, we provided a preliminary characterization of the gut microbiota and mycobiota dynamics in a small cohort of patients with pneumonia-derived sepsis. Despite the modest sample size, our multi-dimensional observations hint at a degree of taxonomic and topological resilience in the global microbial community during the first week of ICU admission, even under the selective pressure of intensive interventions such as broad-spectrum antibiotics. Notably, outcome-based stratification unmasked tentative patterns of prognosis-specific modular rewiring that remained obscured at the community-wide level. We observed that while certain intra-modular microbiota hubs like *Mediterraneibacter* showed potential direct associations with systemic inflammation, fungal signals—typified by *Candida*—appeared to link with clinical parameters primarily through their embedding within cross-kingdom sub-networks rather than through independent abundance shifts. This transition from macro-stability to localized modular reconfiguration suggests that ecological connectivity, particularly at the microbiota-fungal interface, may track with clinical trajectory more closely than traditional diversity metrics. These findings offer preliminary, hypothesis-generating insights into the relationship between the microbial interactome and clinical outcomes in septic patients.

At the community level, the gut microbiota and mycobiota exhibited remarkable resilience during the first week of ICU admission. Both alpha - and beta-diversity remained consistent across groups despite severe septic stress, echoing observations by Mu et al. [31] that global indices often exhibit relative inertia in early critical illness. While our cohort displayed a taxonomic profile typical of sepsis-associated dysbiosis—dominated by *Bacillota*, *Bacteroidota*, and *Pseudomonadota* [32–35]—no significant compositional shifts were identified post-treatment. This stability contrasts with some prior reports of rapid genus-level fluctuations. This discrepancy may stem from our use of ALDEx2, a conservative compositional framework [36]. Unlike traditional tools like LEfSe or STAMP, which often analyze raw proportions and may yield spurious correlations, ALDEx2 accounts for the mathematical interdependence and uncertainty of sequencing data. By minimizing false positives, our approach underscores a conserved taxonomic landscape in this septic cohort despite intensive clinical interventions.

As a critical but often underappreciated component of the gut ecosystem, the mycobiota’s community dynamics carry significant biological implications across various disease contexts [37–39]. In our longitudinal assessment, the mycobiota exhibited more perceptible fluctuations compared to the relative inertia of the microbiota. While global fungal alpha- and beta-diversity remained stable—consistent with findings in severe acute pancreatitis [40]—outcome-based stratification revealed a significant increase in fungal richness (Chao1) specifically in the poor-outcome group (BC vs. BT). This selective rise, likely driven by the recruitment of rare fungal taxa, may reflect a compromised intestinal microbial barrier, where weakened colonization resistance allows for the transient emergence of opportunistic fungi. Similar mycobiota shifts have been noted in COVID-19 [41], where diversity trajectories often parallel host recovery. However, the lack of significant correlation between Chao1 changes and clinical markers (SOFA or cytokines) suggests that shifts in fungal richness—particularly involving rare taxa—may not be linearly synchronized with clinical severity in the acute phase of sepsis. These microecological deviations likely reflect complex systemic fluctuations that warrant further investigation in larger cohorts.

Beyond the observed fluctuations in fungal richness, our network analysis hinted at a more subtle structural reorganization within the inter-kingdom interactome. A noteworthy observation was the emergence of ModM1, a cross-kingdom assembly unique to the favorable outcome group, appearing even as certain global topological metrics—such as the interdomain ratio and mean degree—showed a downward trend. Within this framework, the hub genus *Mediterraneibacter* (formerly classified under the *Ruminococcus* genus) exhibited a negative correlation with systemic inflammation, a potential clinical relevance that appeared independent of its global network role. While *Mediterraneibacter* is often discussed as a putative pathobiont in chronic inflammatory contexts—where it may potentially aggravate mucosal injury by degrading intestinal mucus and triggering excessive immune responses [42]—recent evidence suggests its functional impact may be highly context-dependent [43, 44]. In the acute setting of sepsis, its significance as a prevalent obligate anaerobe has been increasingly noted; for instance, the maintenance of an ‘anaerobic barrier’ is considered potentially helpful for limiting systemic inflammation during empirical antibiotic therapy [45]. The formation of this *Mediterraneibacter*-centered module might represent a tentative structural transition toward gut homeostatic restoration.

We speculate that *Mediterraneibacter*—possibly through metabolite production (e.g., SCFAs) or ecological co-occupancy—could contribute to supporting immune balance and barrier integrity [42–45]. This is consistent with the negative associations observed between its intra-modular abundance and markers like PCT or TNF-α, hinting that its clinical significance might be linked to its integration within specific modular structures.

Furthermore, our cross-kingdom analysis reveals that the mycobiota’s role is primarily ‘context-dependent’. While *Candida* abundance remained stable, its integration into the microbiota-led module ModM8 correlated positively with IL-8. This synergistic influence suggests that fungi affect host inflammation through inter-kingdom embedding rather than independent dominance. Such deep integration offers a tentative explanation for why antifungal overtreatment can exacerbate organ injury and increase mortality [46, 47]. Disrupting intestinal fungi may inadvertently dismantle protective microbiota frameworks anchored within these cross-kingdom architectures. Additionally, the identification of the *Blumeria*-associated ModM4 in the BC group—which showed no clinical correlation—likely reflects population-specific biogeography rather than active pathogenesis [48]. Collectively, these observations underscore the importance of isolating truly disease-relevant taxa from environment-specific background signals.

In summary, our observations suggest that the early phase of sepsis may be characterized by a subtle rewiring of cross-kingdom association patterns, even when overt compositional shifts are not yet prominent. This observed precedence of network reorganization hints at the possibility that interaction dynamics could serve as early indicators of ecosystem flux, whereas detectable shifts in relative abundance might require a longer temporal window to manifest [49].

While the “gut-lung axis” has been confirmed to be extensively involved in the pathological process of sepsis through various bidirectional mechanisms, effective intervention measures for gut microbiota homeostasis remain unclear. Previous research paradigms have mostly focused on how quantitative changes in lung and gut microbes (such as the overgrowth of pathogens) drive infection progression [18, 50–52]. However, our longitudinal observations, alongside other recent studies [31–35], found that despite the severe deterioration of clinical symptoms, microbial abundance and global diversity showed preliminary signs of relative stability in the short term. This discrepancy may suggest that taxonomic apparent stability masks deeper ecological succession to some extent, such as the changes in co-occurrence network relationships identified in our study.

Research by Narayana et al. on the dynamics of gut-lung microecology in patients with bronchiectasis provides an important reference: in these patients, although the overlap of microbial species and quantities between the lung and gut is extremely low, their unique trans-kingdom interactome shows a significant correlation with clinical severity[53]. This offers the possibility that changes in the co-occurrence network itself may also be an independent and important component of the dysregulation of “gut-lung axis”. Based on these limited observations, we speculate that the rearrangement of gut microbial interrelationships in early sepsis may precede obvious numerical fluctuations, which might be a more sensitive potential signal of gut-lung homeostasis dysregulation than abundance metrics, reflecting or even participating in the disease progression process.

Given that the topological remodeling observed in this study tends to manifest as local perturbations within specific ecological niches rather than global network collapses, we shifted our perspective from isolated species to the exploratory concept of “functional modules”. These preliminary insights suggest a potential shift in the direction of clinical translation: from broad-spectrum interventions like FMT toward “module-targeted approaches”. Integrated with the concept of “Precision Prebiotics” [54], we hypothesize that selectively supporting core functional clusters (e.g., the *Mediterraneibacter*-centric ModM1) could reinforce gut-lung homeostasis more effectively than targeting isolated species. Theoretically, this modular restoration could consolidate the fragile ecological framework in septic patients. While these results warrant further validation in larger, multi-center studies, they underscore the value of sub-modular analysis in unmasking microbial-host interactions within the heterogeneous landscape of sepsis.

### Limitations

Despite the distinctive insights provided by this integrated cross-kingdom analysis, several limitations warrant consideration. First, this is a single-center, small exploratory cohort with only 14 patients, leading to limited statistical power and an elevated risk of Type II errors, which may obscure subtle but biologically relevant associations between microbial features and clinical outcomes. Second, the relatively short 7-day longitudinal sampling window only captures early-phase dynamics; long-term microbial succession and its links to late outcomes, including 28-day mortality and post-sepsis complications, remain uncharacterized. Third, given the extreme pathological stress of sepsis, microbial functional output often undergoes dramatic shifts that decouple from taxonomic identity; this renders traditional amplicon-based functional predictive models less clinically meaningful, and thus, we refrained from performing amplicon-based functional prediction. Fourth, detailed information on antibiotic timing, dosage, duration, and cumulative exposure was not fully captured in this study; such antimicrobial pressure is a major confounder that can independently reshape both gut microbiota and mycobiota structure. Fifth, no antifungal agents were administered during the study period, so the impact of antifungal therapy on fungal community dynamics and trans-kingdom interactions could not be evaluated. Furthermore, the current scarcity of comprehensive functional databases and specialized bioinformatic tools for human gut fungi remains a major bottleneck; this deficit restricts our ability to fully describe the cross-kingdom functional landscape, even with the potential support of multi-omic data. Sixth, co-occurrence network analysis only reveals topological correlations rather than direct ecological or causal interactions; true microbial cross-talk requires validation by culturomics or metabolomic studies. Seventh, fecal sampling provides a surrogate profile of the distal gut microbiota and may not fully reflect spatial heterogeneity or in situ crosstalk along the gut–lung axis in sepsis. Finally, as an observational study, this work cannot establish direct causality, and the sample scale also restricted our capacity for high‑dimensional analysis of complex host‑microbe interactions.

## Conclusion

In conclusion, our preliminary findings hint that in the early phase of pneumonia-derived sepsis, the gut microbiome appears to maintain relative taxonomic stability alongside subtle, module-specific reorganization of inter-kingdom interaction patterns. Within this small cohort, microecological evolution appeared to manifest primarily as adjustments in ecological connectivity rather than overt shifts in microbial abundance, with these structural changes potentially paralleling aspects of clinical progression.

Notably, hub-anchored modular configurations (*Mediterraneibacter*-centered ModM1 and fungal-integrated ModM8) showed tentative associations with inflammatory trajectories. These observations suggest that microbial effects may be more closely linked to topological embedding within cross-kingdom networks than to absolute abundance. In contrast, other modules, such as the *Blumeria*-centered ModM4, may represent relatively stable ecological backgrounds rather than active pathological drivers.

Given the limited sample size, these associations should be interpreted cautiously. Nevertheless, this study offers a preliminary topological framework for understanding sepsis-related microecological dynamics and provides exploratory insights that require validation regarding ecological connectivity and trans-kingdom integration as potential dimensions for evaluating microbial alterations in critically ill patients. Larger, multi-center longitudinal studies are required to validate and refine these observations.

## Supplementary Information

Supplementary Material 1

## Data Availability

The sequencing data generated in this study have been deposited in the NCBI Sequence Read Archive (SRA) under BioProject accession number PRJNA1326178. For any additional information or specific data requests, interested researchers may contact the corresponding author.

## Abbreviations

B/F: Bacteroidetes to Firmicutes ratio
CLIF: Common Longitudinal ICU data Format
ICU: Intensive Care Unit
SOFA score: Sequential Organ Failure Assessment score
APACHE II score: Acute Physiology and Chronic Health Evaluation score
CRP: C-reactive protein
PCT: Procalcitonin
ASV: Amplicon sequence variants
TSS: Total sum scaling
PCoA: Principal Coordinate Analysis
PERMANOVA: Permutational Multivariate Analysis of Variance
CLR: Centered log-ratio
Rmcorr: Repeated-measures correlations
BALF: Bronchoalveolar lavage fluid
mNGS: Metagenomic next-generation sequencing
MDROs: Multidrug-resistant organisms
LEfSe: Linear discriminant analysis effect size
COVID-19: Coronavirus disease 2019
