# Supplementary material for: Longitudinal dynamics of gut microbiota and mycobiota in pneumonia-derived sepsis: evidence of taxonomic stability and trans-kingdom network reorganization
Source: Gut Pathog. 2026 May 30;18:65. doi: 10.1186/s13099-026-00838-0 (PMC13459335; doi:10.1186/s13099-026-00838-0)
Supplement: Supplementary file 1 [file 13099_2026_838_MOESM1_ESM.docx]

**Supplementary Document**

**Supplementary Figure 1. Flowchart of patient enrollment and exclusion criteria in the pneumonia-derived sepsis cohort.**

**Supplementary Figure 2. Summary of pathogen detection methods and antibiotic usage in pneumonia-derived sepsis patients.**

**(A)** Distribution of clinical sample types used for pathogen detection, including bronchoalveolar lavage fluid (BALF), blood, or absence of microbiological sampling.

**(B)** Results of culture-based pathogen identification, showing the detected microbiota species and culture-negative cases.

**(C)** Gram stain classification of identified pathogens, categorized as Gram-positive, Gram-negative, or negative.

**(D)** Results of metagenomic next-generation sequencing (mNGS)-based pathogen detection, including identified organisms and negative findings.

**(E)** Categories of antibiotics administered during the 7-day study period, summarized by major antibiotic classes or combinations.

**Supplementary Figure 3. Alpha diversity analyses of gut microbiota and fungal communities stratified by clinical outcome.**

**(A-F)** Alpha diversity indices of the gut bacteriome in patients with good outcomes (GC vs. GT) and poor outcomes (BC vs. BT), including Chao1 richness (A), dominance (B), Good’s coverage (C), Simpson index (D), Pielou’s evenness (E), and Shannon diversity (F).

**(G-L)** Alpha diversity indices of the gut mycobiome across the same outcome-stratified comparisons, including Chao1 richness (G), dominance (H), Good’s coverage (I), Simpson index (J), Pielou’s evenness (K), and Shannon diversity (L). A significant increase in Chao1 richness was observed after treatment in the poor-outcome group (BC vs. BT), whereas other diversity metrics remained unchanged, indicating an expansion primarily driven by low-abundance fungal taxa.

**Supplementary Figure 4. Paired comparison and between-group analysis of microbial network topological properties.**

**(A-I)** Paired comparisons of key network attributes derived from the co-occurrence networks. Each line represents paired samples from the same patient across time points.

**(J-N)** Between-group comparisons of selected topological parameters. Statistical significance between groups is indicated where applicable. Network metrics were calculated from genus-level co-occurrence networks constructed using significant correlations as described in the Methods.

**Supplementary Figure 5. Clinical correlations of GT-specific module hubs across the entire cohort.**

Spearman correlation heatmap between the relative abundances of the four module hubs (identified from the GT-specific ModM1 module) and clinical parameters across all study participants.

**Supplementary Figure 6. Clinical correlations of key module hubs within the GT group.**

Spearman correlations between the relative abundances of the four specific module hubs identified within the GT-group-specific ModM1 module and clinical parameters.

**Supplementary Figure 7. Clinical relevance of the fungal-integrated ModM8 module in the GT group.**

Spearman correlation analysis between the aggregate relative abundance of the ModM8 module and clinical parameters within the GT group.

**Supplementary Figure 8. Clinical correlations of intra-modular Candida in the GT group.**

Spearman correlation analysis between the relative abundance of intra-modular Candida (the core genus of ModM8) and clinical parameters within the GT group.

**Supplementary Table 1.** Repeated measures correlation between longitudinal changes in gut mycobiota richness (Chao1 index) and clinical metrics in pneumonia-derived septic patients with bad outcomes (BC to BT).

| **Variable** | **R** | ***P*-value** |
| --- | --- | --- |
| SOFA | 0.166 | 0.754 |
| PCT | -0.797 | 0.058 |
| CRP | -0.429 | 0.396 |
| IL-8 | -0.301 | 0.562 |
| IL-1β | 0.199 | 0.706 |
| TNF-α | -0.124 | 0.815 |
| IL-10 | 0.099 | 0.851 |
| IL-6 | -0.073 | 0.891 |

Notes. BC, bad-outcome group at Day 1 (baseline); BT, bad-outcome group at Day 7 (after seven days of treatment); IL, interleukin; TNF-α, tumor necrosis factor-α; SOFA, Sequential Organ Failure Assessment; CRP, C-reactive protein; PCT, procalcitonin.

**Supplementary Table 2.** Spearman correlation between gut mycobiota richness (Chao1 index) and clinical parameters of the BT group.

| **Variable** | **R** | ***P*-value** |
| --- | --- | --- |
| SOFA | 0.600 | 0.285 |
| CRP | 0.600 | 0.285 |
| PCT | 0.500 | 0.391 |
| IL-6 | 0.300 | 0.624 |
| TNF-α | -0.200 | 0.747 |
| IL-10 | 0.051 | 0.935 |
| IL-8 | 0.000 | 1.000 |
| IL-1β | 0.783 | 0.118 |

Notes. BT, bad-outcome group at Day 7 (after seven days of treatment); SOFA, Sequential Organ Failure Assessment; CRP, C-reactive protein; PCT, procalcitonin; IL, interleukin; TNF-α, tumor necrosis factor-α.

**Supplementary Table 3.** Comparison of topological metrics of microbial co-occurrence networks between AC and AT in pneumonia-derived septic patients.

| **Metric** | **AC Mean** | **AT Mean** | **Mean Difference** | ***P*-value** |
| --- | --- | --- | --- | --- |
| Number of Edges | 15.857 | 11.714 | -4.143 | 0.281 |
| Average Path Length | 1.584 | 1.480 | -0.103 | 0.387 |
| Average Degree | 2.423 | 2.168 | -0.255 | 0.455 |
| Betweenness Centralization | 0.133 | 0.114 | -0.018 | 0.605 |
| Network Density | 0.260 | 0.242 | -0.018 | 0.638 |
| Connectance | 0.260 | 0.242 | -0.018 | 0.638 |
| Number of Nodes | 10.143 | 9.714 | -0.429 | 0.765 |
| Clustering Coefficient | 0.533 | 0.518 | -0.015 | 0.822 |
| Modularity | 0.144 | 0.178 | 0.034 | 0.972 |

Notes. AC, baseline group (Day 1); AT, post-treatment group (Day 7).

**Supplementary Table 4.** Spearman correlation between the total abundance of module ModM4 and clinical parameters in the BC group.

| **Variable** | **R** | ***P*-value** |
| --- | --- | --- |
| SOFA | -0.726 | 0.165 |
| TNF-α | 0.707 | 0.182 |
| IL-10 | -0.707 | 0.182 |
| CRP | 0.544 | 0.343 |
| IL-6 | 0.354 | 0.559 |
| PCT | 0.354 | 0.559 |
| IL-8 | 0.000 | 1.000 |
| IL-1β | 0.000 | 1.000 |

Notes. BC, bad-outcome group at Day 1 (baseline); SOFA, Sequential Organ Failure Assessment; CRP, C-reactive protein; PCT, procalcitonin; IL, interleukin; TNF-α, tumor necrosis factor-α.

**Supplementary Table 5.** Spearman correlation between the intra-modular relative abundance of Blumeria (a core member of ModM4) and clinical parameters in the BC group.

| **Variable** | **R** | ***P*-value** |
| --- | --- | --- |
| SOFA | -0.725 | 0.165 |
| IL-6 | 0.354 | 0.559 |
| IL-8 | 0.000 | 1.000 |
| TNF-α | 0.707 | 0.182 |
| IL-1β | 0.000 | 1.000 |
| IL-10 | -0.707 | 0.182 |
| PCT | 0.354 | 0.559 |
| CRP | 0.544 | 0.343 |

Notes. BC, bad-outcome group at Day 1 (baseline); IL, interleukin; TNF-α, tumor necrosis factor-α; SOFA, Sequential Organ Failure Assessment; CRP, C-reactive protein; PCT, procalcitonin.
